# Highly Pathogenic Avian Influenza A(H5N1) Clade 2.3.4.4b Virus Infection in Domestic Dairy Cattle and Cats, United States, 2024

**DOI:** 10.3201/eid3007.240508

**Published:** 2024-07

**Authors:** Eric R. Burrough, Drew R. Magstadt, Barbara Petersen, Simon J. Timmermans, Phillip C. Gauger, Jianqiang Zhang, Chris Siepker, Marta Mainenti, Ganwu Li, Alexis C. Thompson, Patrick J. Gorden, Paul J. Plummer, Rodger Main

**Affiliations:** Iowa State University College of Veterinary Medicine, Ames, Iowa, USA (E.R. Burrough, D.R. Magstadt, P.C. Gauger, J. Zhang, C. Siepker, M. Mainenti, G. Li, P.J. Gorden, P.J. Plummer, R. Main);; Sunrise Veterinary Service PLLC, Amarillo, Texas, USA (B. Petersen);; Veterinary Research & Consulting Services LLC, Hays, Kansas, USA (S.J. Timmermans);; Texas A&M Veterinary Medical Diagnostic Laboratory, College Station, Texas, USA (A.C. Thompson)

**Keywords:** influenza, highly pathogenic avian influenza A(H5N1), HPAI, avian influenza, H5N1, clade 2.3.4.4b, bovine influenza A, dairy cattle, cats, spillover, respiratory infections, viruses, zoonoses, United States

## Abstract

We report highly pathogenic avian influenza A(H5N1) virus in dairy cattle and cats in Kansas and Texas, United States, which reflects the continued spread of clade 2.3.4.4b viruses that entered the country in late 2021. Infected cattle experienced nonspecific illness, reduced feed intake and rumination, and an abrupt drop in milk production, but fatal systemic influenza infection developed in domestic cats fed raw (unpasteurized) colostrum and milk from affected cows. Cow-to-cow transmission appears to have occurred because infections were observed in cattle on Michigan, Idaho, and Ohio farms where avian influenza virus–infected cows were transported. Although the US Food and Drug Administration has indicated the commercial milk supply remains safe, the detection of influenza virus in unpasteurized bovine milk is a concern because of potential cross-species transmission. Continued surveillance of highly pathogenic avian influenza viruses in domestic production animals is needed to prevent cross-species and mammal-to-mammal transmission.

Highly pathogenic avian influenza (HPAI) viruses pose a threat to wild birds and poultry globally, and HPAI H5N1 viruses are of even greater concern because of their frequent spillover into mammals. In late 2021, the Eurasian strain of H5N1 (clade 2.3.4.4b) was detected in North America (*1*,*2*) and initiated an outbreak that continued into 2024. Spillover detections and deaths from this clade have been reported in both terrestrial and marine mammals in the United States (*3*,*4*). The detection of HPAI H5N1 clade 2.3.4.4b virus in severe cases of human disease in Ecuador (*5*) and Chile (*6*) raises further concerns regarding the pandemic potential of specific HPAI viruses.

In February 2024, veterinarians were alerted to a syndrome occurring in lactating dairy cattle in the panhandle region of northern Texas. Nonspecific illness accompanied by reduced feed intake and rumination and an abrupt drop in milk production developed in affected animals. The milk from most affected cows had a thickened, creamy yellow appearance similar to colostrum. On affected farms, incidence appeared to peak 4–6 days after the first animals were affected and then tapered off within 10–14 days; afterward, most animals were slowly returned to regular milking. Clinical signs were commonly reported in multiparous cows during middle to late lactation; ≈10%–15% illness and minimal death of cattle were observed on affected farms. Initial submissions of blood, urine, feces, milk, and nasal swab samples and postmortem tissues to regional diagnostic laboratories did not reveal a consistent, specific cause for reduced milk production. Milk cultures were often negative, and serum chemistry testing showed mildly increased aspartate aminotransferase, gamma-glutamyl transferase, creatinine kinase, and bilirubin values, whereas complete blood counts showed variable anemia and leukocytopenia. 

In early March 2024, similar clinical cases were reported in dairy cattle in southwestern Kansas and northeastern New Mexico; deaths of wild birds and domestic cats were also observed within affected sites in the Texas panhandle. In >1 dairy farms in Texas, deaths occurred in domestic cats fed raw colostrum and milk from sick cows that were in the hospital parlor. Antemortem clinical signs in affected cats were depressed mental state, stiff body movements, ataxia, blindness, circling, and copious oculonasal discharge. Neurologic exams of affected cats revealed the absence of menace reflexes and pupillary light responses with a weak blink response.

On March 21, 2024, milk, serum, and fresh and fixed tissue samples from cattle located in affected dairies in Texas and 2 deceased cats from an affected Texas dairy farm were received at the Iowa State University Veterinary Diagnostic Laboratory (ISUVDL; Ames, IA, USA). The next day, similar sets of samples were received from cattle located in affected dairies in Kansas. Milk and tissue samples from cattle and tissue samples from the cats tested positive for influenza A virus (IAV) by screening PCR, which was confirmed and characterized as HPAI H5N1 virus by the US Department of Agriculture National Veterinary Services Laboratory. Detection led to an initial press release by the US Department of Agriculture Animal and Plant Health Inspection Service on March 25, 2024, confirming HPAI virus in dairy cattle (*7*). We report the characterizations performed at the ISUVDL for HPAI H5N1 viruses infecting cattle and cats in Kansas and Texas.

## Materials and Methods

Milk samples (cases 2–5) and fresh and formalin-fixed tissues (cases 1, 3–5) from dairy cattle were received at the ISUVDL from Texas on March 21 and from Kansas on March 22, 2024. The cattle exhibited nonspecific illness and reduced lactation, as described previously. The tissue samples for diagnostic testing came from 3 cows that were euthanized and 3 that died naturally; all postmortem examinations were performed on the premises of affected farms.

The bodies of 2 adult domestic shorthaired cats from a north Texas dairy farm were received at the ISUVDL for a complete postmortem examination on March 21, 2024. The cats were found dead with no apparent signs of injury and were from a resident population of ≈24 domestic cats that had been fed milk from sick cows. Clinical disease in cows on that farm was first noted on March 16; the cats became sick on March 17, and several cats died in a cluster during March 19–20. In total, >50% of the cats at that dairy became ill and died. We collected cerebrum, cerebellum, eye, lung, heart, spleen, liver, lymph node, and kidney tissue samples from the cats and placed them in 10% neutral-buffered formalin for histopathology.

At ISUVDL, we trimmed, embedded in paraffin, and processed formalin-fixed tissues from affected cattle and cats for hematoxylin/eosin staining and histologic evaluation. For immunohistochemistry (IHC), we prepared 4-µm–thick sections from paraffin-embedded tissues, placed them on Superfrost Plus slides (VWR, https://www.vwr.com), and dried them for 20 minutes at 60°C. We used a Ventana Discovery Ultra IHC/ISH research platform (Roche, https://www.roche.com) for deparaffinization until and including counterstaining. We obtained all products except the primary antibody from Roche. Automated deparaffination was followed by enzymatic digestion with protease 1 for 8 minutes at 37°C and endogenous peroxidase blocking. We obtained the primary influenza A virus antibody from the hybridoma cell line H16-L10–4R5 (ATCC, https://www.atcc.org) and diluted at 1:100 in Discovery PSS diluent; we incubated sections with antibody for 32 minutes at room temperature. Next, we incubated the sections with a hapten-labeled conjugate, Discovery anti-mouse HQ, for 16 minutes at 37°C followed by a 16-minute incubation with the horseradish peroxidase conjugate, Discovery anti-HQ HRP. We used a ChromoMap DAB kit for antigen visualization, followed by counterstaining with hematoxylin and then bluing. Positive controls were sections of IAV-positive swine lung. Negative controls were sections of brain, lung, and eyes from cats not infected with IAV.

We diluted milk samples 1:3 vol/vol in phosphate buffered saline, pH 7.4 (Gibco/Thermo Fisher Scientific, https://www.thermofisher.com) by mixing 1 unit volume of milk and 3 unit volumes of phosphate buffered saline. We prepared 10% homogenates of mammary glands, brains, lungs, spleens, and lymph nodes in Earle’s balanced salt solution (Sigma-Aldrich, https://www.sigmaaldrich.com). Processing was not necessary for ocular fluid, rumen content, or serum samples. After processing, we extracted samples according to a National Animal Health Laboratory Network (NAHLN) protocol that had 2 NAHLN-approved deviations for ISUVDL consisting of the MagMax Viral RNA Isolation Kit for 100 µL sample volumes and a Kingfisher Flex instrument (both Thermo Fisher Scientific).

We performed real-time reverse transcription PCR (rRT-PCR) by using an NAHLN-approved assay with 1 deviation, which was the VetMAX-Gold SIV Detection kit (Thermo Fisher Scientific), to screen for the presence of IAV RNA. We tested samples along with the VetMAX XENO Internal Positive Control to monitor the possible presence of PCR inhibitors. Each rRT-PCR 96-well plate had 2 positive amplification controls, 2 negative amplification controls, 1 positive extraction control, and 1 negative extraction control. We ran the rRT-PCR on an ABI 7500 Fast thermocycler and analyzed data with Design and Analysis Software 2.7.0 (both Thermo Fisher Scientific). We considered samples with cycle threshold (Ct) values <40.0 to be positive for virus.

After the screening rRT-PCR, we analyzed IAV RNA–positive samples for the H5 subtype and H5 clade 2.3.4.4b by using the same RNA extraction and NAHLN-approved rRT-PCR protocols as described previously, according to standard operating procedures. We performed PCR on the ABI 7500 Fast thermocycler by using appropriate controls to detect H5-specific IAV. We considered samples with Ct values <40.0 to be positive for the IAV H5 subtype.

We conducted genomic sequencing of 2 milk samples from infected dairy cattle from Texas and 2 tissue samples (lung and brain) from cats that died at a different Texas dairy. We subjected the whole-genome sequencing data to bioinformatics analysis to assemble the 8 different IAV segment sequences according to previously described methods (*8*). We used the hemagglutinin (HA) and neuraminidase (NA) sequences for phylogenetic analysis. We obtained reference sequences for the HA and NA segments of IAV H5 clade 2.3.4.4 from publicly available databases, including GISAID (https://www.gisaid.org) and GenBank. We aligned the sequences by using MAFFT version 7.520 software (https://mafft.cbrc.jp/alignment/server/index.html) to create multiple sequence alignments for subsequent phylogenetic analysis. We used IQTree2 (https://github.com/iqtree/iqtree2) to construct the phylogenetic tree from the aligned sequences. The software was configured to automatically identify the optimal substitution model by using the ModelFinder Plus option, ensuring the selection of the most suitable model for the dataset and, thereby, improving the accuracy of the reconstructed tree. We visualized the resulting phylogenetic tree by using iTOL (https://itol.embl.de), a web-based platform for interactive tree exploration and annotation.

## Results

### Gross Lesions in Cows and Cats

All cows were in good body condition with adequate rumen fill and no external indications of disease. Postmortem examinations of the affected dairy cows revealed firm mammary glands typical of mastitis; however, mammary gland lesions were not consistent. Two cows that were acutely ill before postmortem examination had grossly normal milk and no abnormal mammary gland lesions. The gastrointestinal tract of some cows had small abomasal ulcers and shallow linear erosions of the intestines, but those observations were also not consistent in all animals. The colon contents were brown and sticky, suggesting moderate dehydration. The feces contained feed particles that appeared to have undergone minimal ruminal fermentation. The rumen contents had normal color and appearance but appeared to have undergone minimal fermentation.

The 2 adult cats (1 intact male, 1 intact female) received at the ISUVDL were in adequate body and postmortem condition. External examination was unremarkable. Mild hemorrhages were observed in the subcutaneous tissues over the dorsal skull, and multifocal meningeal hemorrhages were observed in the cerebrums of both cats. The gastrointestinal tracts were empty, and no other gross lesions were observed. 

### Microscopic Lesions in Cows and Cats

**Figure 1:**
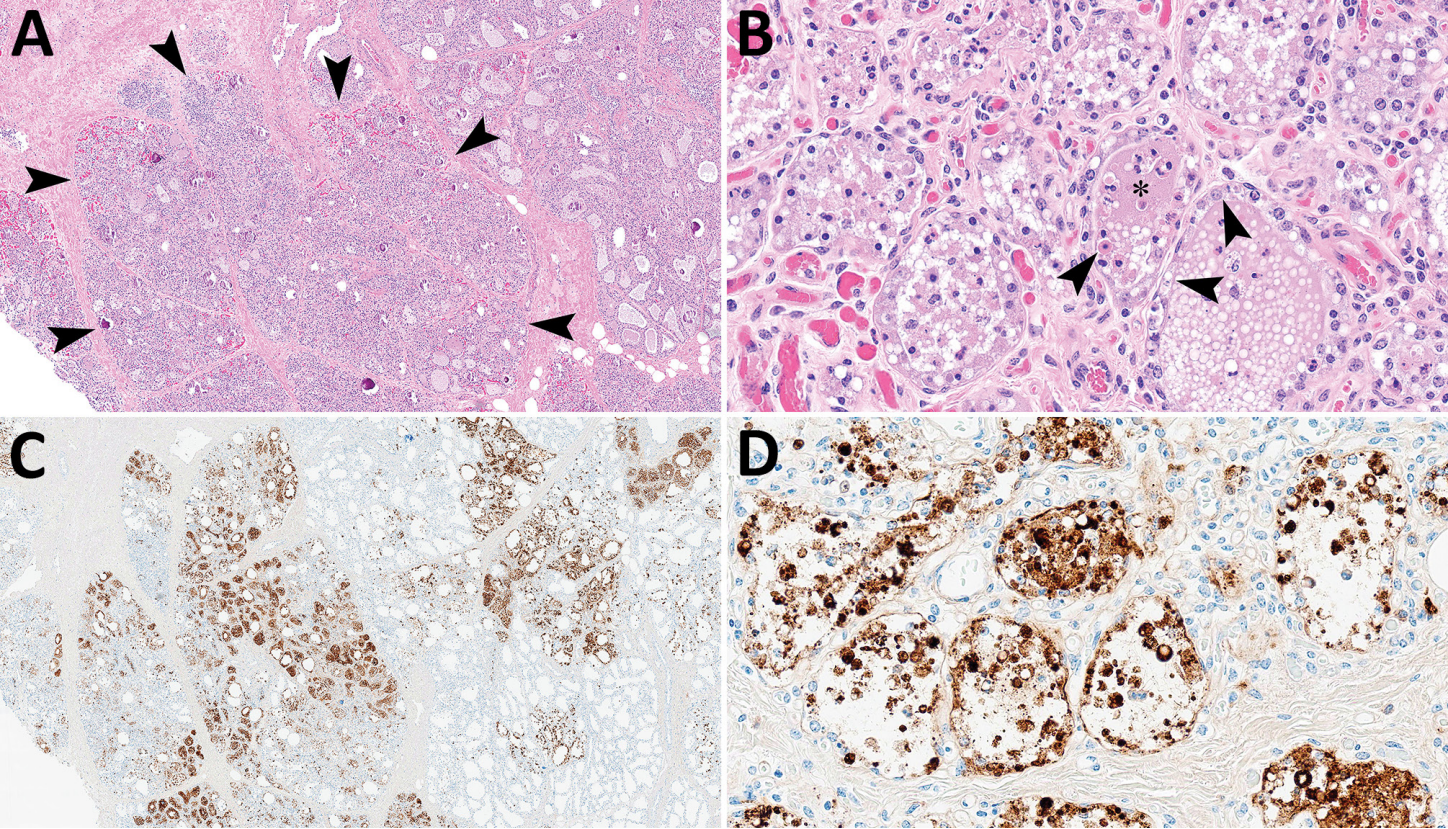
Mammary gland lesions in cattle in study of highly pathogenic avian influenza A(H5N1) clade 2.3.4.4b virus infection in domestic dairy cattle and cats, United States, 2024. A, B) Mammary gland tissue sections stained with hematoxylin and eosin. A) Arrowheads indicate segmental loss within open secretory mammary alveoli. Original magnification ×40. B) Arrowheads indicate epithelial degeneration and necrosis lining alveoli with intraluminal sloughing. Asterisk indicates intraluminal neutrophilic inflammation. Original magnification ×400. C, D) Mammary gland tissue sections stained by using avian influenza A immunohistochemistry. C) Brown staining indicates lobular distribution of avian influenza A virus. Original magnification ×40. D) Brown staining indicates strong nuclear and intracytoplasmic immunoreactivity of intact and sloughed epithelial cells within mammary alveoli. Original magnification ×400.

The chief microscopic lesion observed in affected cows was moderate acute multifocal neutrophilic mastitis (Figure 1); however, mammary glands were not received from every cow. Three cows had mild neutrophilic or lymphocytic hepatitis. Because they were adult cattle, other observed microscopic lesions (e.g., mild lymphoplasmacytic interstitial nephritis and mild to moderate lymphocytic abomasitis) were presumed to be nonspecific, age-related changes. We did not observe major lesions in the other evaluated tissues. We performed IHC for IAV antigen on all evaluated tissues; the only tissues with positive immunoreactivity were mastitic mammary glands from 2 cows that showed nuclear and cytoplasmic labeling of alveolar epithelial cells and cells within lumina (Figure 1) and multifocal germinal centers within a lymph node from 1 cow (Table 1).

**Table 1:** Microscopic lesions observed in cattle in study of highly pathogenic avian influenza A(H5N1) clade 2.3.4.4b virus infection in domestic dairy cattle and cats, United States, 2024*

Case no.	Animal	US state	Suppurative mastitis	Hepatitis	Abomasitis	IAV-positive tissue†
1	Cow 1	Texas	++	+	+	Mammary gland
1	Cow 2	Texas	++	+	++	Mammary gland
3	Cow 1	Kansas	NA	+	+	NA
3	Cow 2	Kansas	NA	–	+	NA
4	Cow 1	Kansas	NA	–	NA	Lymph node
5	Cow 1	Kansas	NA	–	–	NA

*Avian influenza A(H5N1) virus was detected by using real-time reverse transcription PCR. IAV, influenza A virus; NA, not available; –, negative; +, scant lesions; ++, moderate lesions. †Determined by immunohistochemistry.

**Figure 2:**
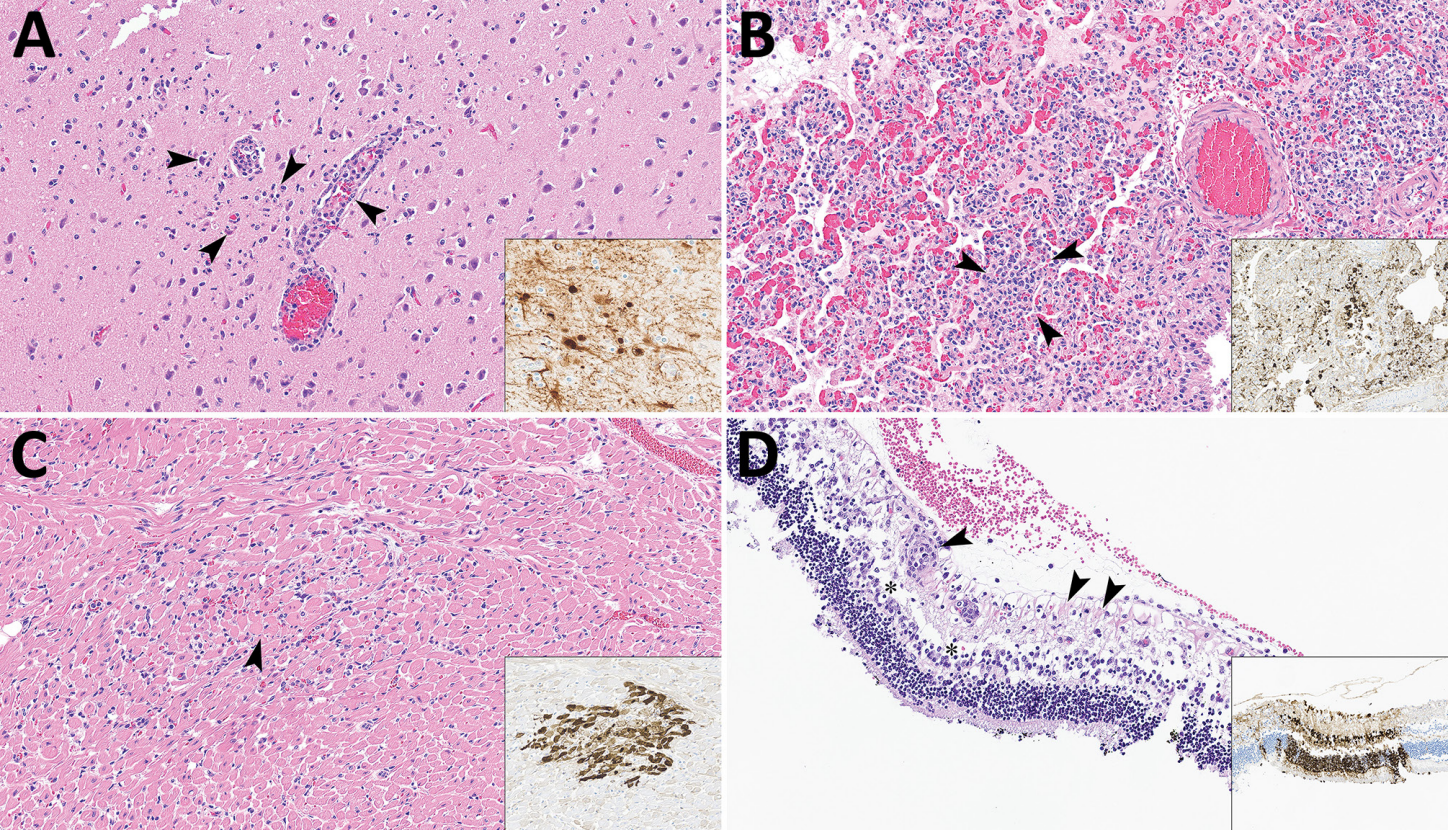
Lesions in cat tissues in study of highly pathogenic avian influenza A(H5N1) clade 2.3.4.4b virus infection in domestic dairy cattle and cats, United States, 2024. Tissue sections were stained with hematoxylin and eosin; insets show brown staining of avian influenza A viruses via immunohistochemistry by using the chromogen 3,3′-diaminobenzidine tetrahydrochloride. Original magnification ×200 for all images and insets. A) Section from cerebral tissue. Arrowheads show perivascular lymphocytic encephalitis, gliosis, and neuronal necrosis. Inset shows neurons. B) Section of lung tissue showing lymphocytic and fibrinous interstitial pneumonia with septal necrosis and alveolar edema; arrowheads indicate lymphocytes. Inset shows bronchiolar epithelium, necrotic cells, and intraseptal mononuclear cells. C) Section of heart tissue. Arrowhead shows interstitial lymphocytic myocarditis and focal peracute myocardial coagulative necrosis. Inset shows cardiomyocytes. D) Section of retinal tissue. Arrowheads show perivascular lymphocytic retinitis with segmental neuronal loss and rarefaction in the ganglion cell layer. Asterisks indicate attenuation of the inner plexiform and nuclear layers with artifactual retinal detachment. Insets shows all layers of the retina segmentally within affected areas have strong cytoplasmic and nuclear immunoreactivity to influenza A virus.

Both cats had microscopic lesions consistent with severe systemic virus infection, including severe subacute multifocal necrotizing and lymphocytic meningoencephalitis with vasculitis and neuronal necrosis, moderate subacute multifocal necrotizing and lymphocytic interstitial pneumonia, moderate to severe subacute multifocal necrotizing and lymphohistiocytic myocarditis, and moderate subacute multifocal lymphoplasmacytic chorioretinitis with ganglion cell necrosis and attenuation of the internal plexiform and nuclear layers (Table 2; Figure 2). We performed IHC for IAV antigen on multiple tissues (brain, eye, lung, heart, spleen, liver, and kidney). We detected positive IAV immunoreactivity in brain (intracytoplasmic, intranuclear, and axonal immunolabeling of neurons), lung, and heart, and multifocal and segmental immunoreactivity within all layers of the retina (Figure 2).

**Table 2:** Microscopic lesions observed in cats in study of highly pathogenic avian influenza A(H5N1) clade 2.3.4.4b virus infection in domestic dairy cattle and cats, United States, 2024*

Case no.	Animal	US state	Encephalitis	Retinitis	Interstitial pneumonia	Necrotizing myocarditis	Vacuolar hepatopathy	IAV-positive tissues†
6	Cat 1	Texas	+++	–	++	++	+	Brain, lung, heart
6	Cat 2	Texas	+++	++	++	++	+	Brain, lung, heart

*Avian influenza A(H5N1) virus was detected by using real-time reverse transcription PCR. IAV, influenza A virus; –, negative; +, scant lesions; ++, moderate lesions; +++, severe lesions. †Determined by immunohistochemistry.

### PCR Data from Cows and Cats

We tested various samples from 8 clinically affected mature dairy cows by IAV screening and H5 subtype-specific PCR (Table 3). Milk and mammary gland homogenates consistently showed low Ct values: 12.3–16.9 by IAV screening PCR, 17.6–23.1 by H5 subtype PCR, and 14.7–20.0 by H5 2.3.4.4 clade PCR (case 1, cow 1; case 2, cows 1 and 2; case 3, cow 1; and case 4, cow 1). We forwarded the samples to the National Veterinary Services Laboratory, which confirmed the virus was an HPAI H5N1 virus strain.

**Table 3:** PCR results from various specimens in study of highly pathogenic avian influenza A(H5N1) clade 2.3.4.4b virus infection in domestic dairy cattle and cats, United States, 2024*

Case no.	Animal	State	IAV PCR test	Ct values for IAV PCR							
				Milk	Mammary gland	Brain	Lung	Spleen	Lymph node	Ocular fluid	Rumen contents
1	Cow 1	Texas	IAV screen	NA	16.2	NA	≥40	NA	NA	NA	NA
			H5 subtype		17.9		NA				
			H5 2.3.4.4		17.8		NA				
1	Cow 2	Texas	IAV screen	NA	NA	NA	32.6	NA	NA	NA	NA
			H5 subtype				36.0				
			H5 2.3.4.4				34.8				
2	Cow 1	Texas	IAV screen	12.3	NA	NA	NA	NA	NA	NA	NA
			H5 subtype	18.8							
			H5 2.3.4.4	14.7							
2	Cow 2	Texas	IAV screen	13.1	NA	NA	NA	NA	NA	NA	NA
			H5 subtype	17.6							
			H5 2.3.4.4	15.1							
3	Cow 1	Kansas	IAV screen	15.2	NA	NA	≥40	≥40	≥40	29.0	31.0
			H5 subtype	19.5			NA	NA	NA	31.2	32.7
			H5 2.3.4.4	17.1			NA	NA	NA	30.9	32.2
3	Cow 2	Kansas	IAV screen	NA	NA	NA	≥40	≥40	NA	≥40	≥40
			H5 subtype				NA	NA		NA	NA
			H5 2.3.4.4				NA	NA		NA	NA
4	Cow 1	Kansas	IAV screen	16.9	NA	NA	33.0	≥40	31.2	≥40	29.4
			H5 subtype	23.1			≥40	NA	38.0	NA	32.4
			H5 2.3.4.4	20.0			≥40	NA	33.9	NA	31.1
5	Cow 1	Kansas	IAV screen	≥40	NA	NA	34.1	29.8	NA	NA	NA
			H5 subtype	NA			37.8	36.1			
			H5 2.3.4.4	NA			37.3	33.9			
6	Cat 1	Texas	IAV screen	NA	NA	9.9	17.4	NA	NA	NA	NA
			H5 subtype			10.7	16.5				
			H5 2.3.4.4			11.9	18.0				
6	Cat 2	Texas	IAV screen	NA	NA	13.5	24.4	NA	NA	NA	NA
			H5 subtype			14.0	23.8				
			H5 2.3.4.4			15.2	24.8				

*Ct values were obtained by using real-time reverse transcription PCR tests for influenza A viruses, the H5 subtype, and H5 clade 2.3.4.4 viruses. Ct, cycle threshold; H5, hemagglutinin 5; IAV, influenza A virus; NA, not available.

When they were available, we also tested tissue homogenates (e.g., lung, spleen, and lymph nodes), ocular fluid, and rumen contents from 6 cows by IAV and H5 subtype-specific PCR (Table 3). However, the PCR findings were not consistent. For example, the tissue homogenates and ocular fluid tested positive in some but not all cows. In case 5, cow 1, the milk sample tested negative by IAV screening PCR, but the spleen homogenate tested positive by IAV screening, H5 subtype, and H5 2.3.4.4 PCR. For 2 cows (case 3, cow 1; and case 4, cow 1) that had both milk and rumen contents available, both samples tested positive for IAV. Nevertheless, all IAV-positive nonmammary gland tissue homogenates, ocular fluid, and rumen contents had markedly elevated Ct values in contrast to the low Ct values for milk and mammary gland homogenate samples.

We tested brain and lung samples from the 2 cats (case 6, cats 1 and 2) by IAV screening and H5 subtype-specific PCR (Table 3). Both sample types were positive by IAV screening PCR; Ct values were 9.9–13.5 for brain and 17.4–24.4 for lung samples, indicating high amounts of virus nucleic acid in those samples. The H5 subtype and H5 2.3.4.4 PCR results were also positive for the brain and lung samples; Ct values were consistent with the IAV screening PCR (Table 3).

### Phylogenetic Analyses

We assembled the sequences of all 8 segments of the HPAI viruses from both cow milk and cat tissue samples. We used the hemagglutinin (HA) and neuraminidase (NA) sequences specifically for phylogenetic analysis to delineate the clade of the HA gene and subtype of the NA gene.

**Figure 3:**
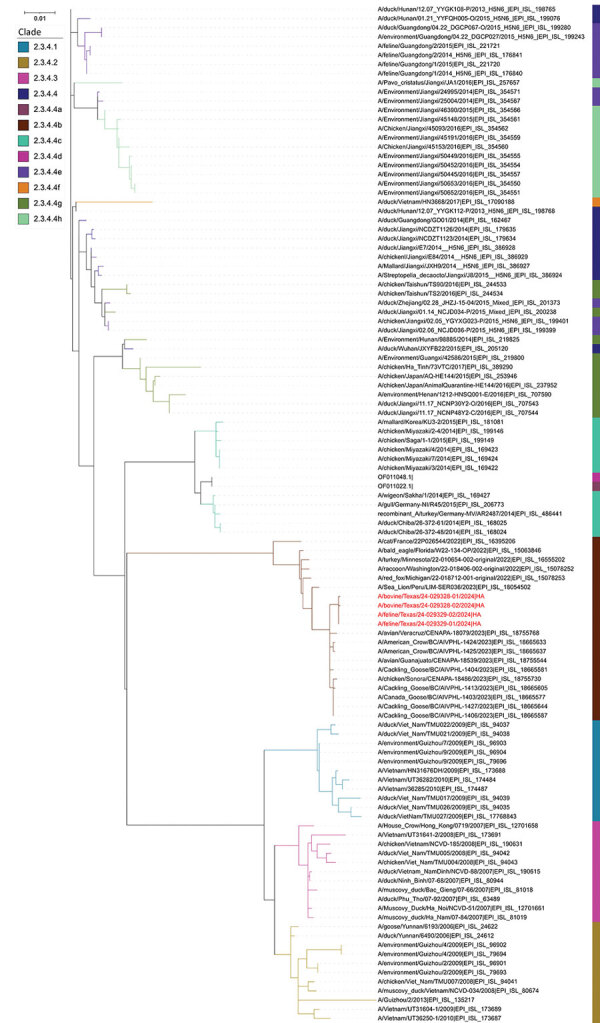
Phylogenetic analysis of hemagglutinin gene sequences in study of highly pathogenic avian influenza A(H5N1) clade 2.3.4.4b virus infection in domestic dairy cattle and cats, United States, 2024. Colors indicate different clades. Red text indicates the virus gene sequences from bovine milk and cats described in this report, confirming those viruses are highly similar and belong to H5 clade 2.3.4.4b. The hemagglutinin sequences from this report are most closely related to A/avian/Guanajuato/CENAPA-18539/2023|EPI_ISL_18755544|A_/_H5 (GISAID, https://www.gisaid.org) and have 99.66%–99.72% nucleotide identities.

For HA gene analysis, both HA sequences derived from cow milk samples exhibited a high degree of similarity, sharing 99.88% nucleotide identity, whereas the 2 HA sequences from cat tissue samples showed complete identity at 100%. The HA sequences from the milk samples had 99.94% nucleotide identities with HA sequences from the cat tissues, resulting in a distinct subcluster comprising all 4 HA sequences, which clustered together with other H5N1 viruses belonging to clade 2.3.4.4b (Figure 3). The HA sequences were deposited in GenBank (accession nos. PP599465 [case 2, cow 1], PP599473 [case 2, cow 2], PP692142 [case 6, cat 1], and PP692195 [case 6, cat 2]).

**Figure 4:**
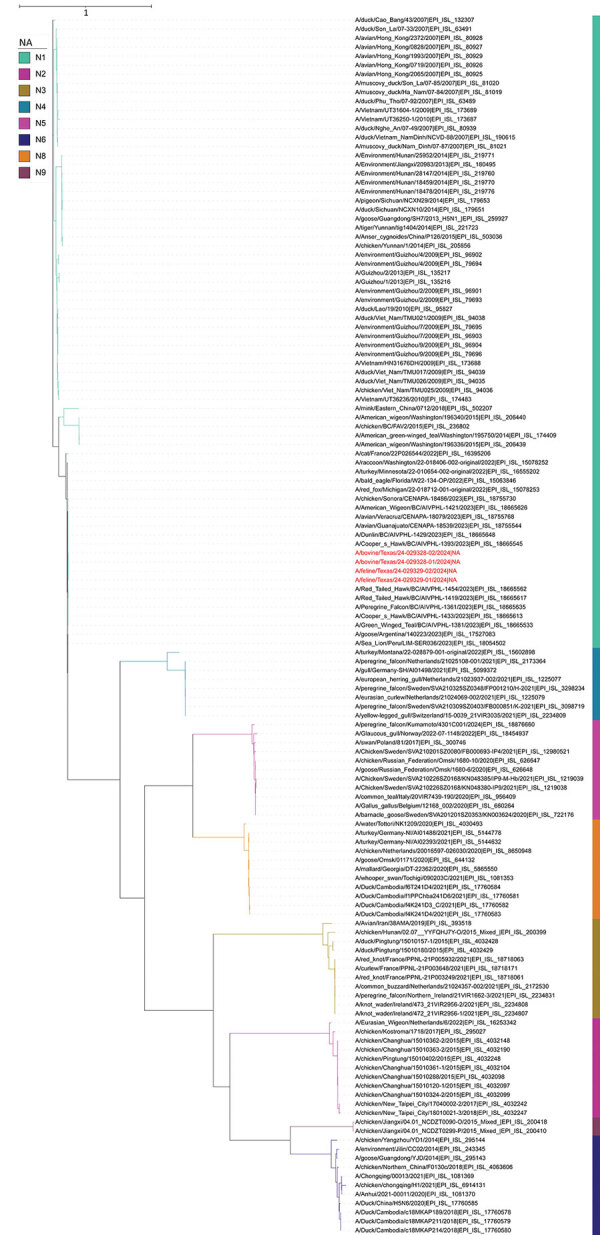
Phylogenetic analysis of neuraminidase gene sequences in study of highly pathogenic avian influenza A(H5N1) clade 2.3.4.4b virus infection in domestic dairy cattle and cats, United States, 2024. Colors indicate different subtypes. Red text indicates the virus gene sequences from bovine milk and cats described in this report, confirming those viruses belong to the N1 subtype. The neuraminidase sequences from this report had 99.52%–99.59% nucleotide identities to sequences from viruses isolated from a chicken and wild birds in 2023.

For NA gene analysis, the 2 NA sequences obtained from cow milk samples showed 99.93% nucleotide identity. Moreover, the NA sequences derived from the milk samples exhibited complete nucleotide identities (100%) with those from the cat tissues. The 4 NA sequences were grouped within the N1 subtype of HPAI viruses (Figure 4). The NA sequences were deposited in GenBank (accession nos. PP599467 [case 2, cow 1], PP599475 [case 2, cow 2], PP692144 [case 6, cat 1], and PP692197 [case 6, cat 2]).

## Discussion

This case series differs from most previous reports of IAV infection in bovids, which indicated cattle were inapparently infected or resistant to infection (*9*). We describe an H5N1 strain of IAV in dairy cattle that resulted in apparent systemic illness, reduced milk production, and abundant virus shedding in milk. The magnitude of this finding is further emphasized by the high death rate (≈50%) of cats on farm premises that were fed raw colostrum and milk from affected cows; clinical disease and lesions developed that were consistent with previous reports of H5N1 infection in cats presumably derived from consuming infected wild birds (*10*–*12*). Although exposure to and consumption of dead wild birds cannot be completely ruled out for the cats described in this report, the known consumption of unpasteurized milk and colostrum from infected cows and the high amount of virus nucleic acid within the milk make milk and colostrum consumption a likely route of exposure. Therefore, our findings suggest cross-species mammal-to-mammal transmission of HPAI H5N1 virus and raise new concerns regarding the potential for virus spread within mammal populations. Horizontal transmission of HPAI H5N1 virus has been previously demonstrated in experimentally infected cats (*13*) and ferrets (*14*) and is suspected to account for large dieoffs observed during natural outbreaks in mink (*15*) and sea lions (*16*). Future experimental studies of HPAI H5N1 virus in dairy cattle should seek to confirm cross-species transmission to cats and potentially other mammals.

Clinical IAV infection in cattle has been infrequently reported in the published literature. The first report occurred in Japan in 1949, where a short course of disease with pyrexia, anorexia, nasal discharge, pneumonia, and decreased lactation developed in cattle (*17*). In 1997, a similar condition occurred in dairy cows in southwest England leading to a sporadic drop in milk production (*18*), and IAV seroconversion was later associated with reduced milk yield and respiratory disease (*19*–*21*). Rising antibody titers against human-origin influenza A viruses (H1N1 and H3N2) were later again reported in dairy cattle in England, which led to an acute fall in milk production during October 2005–March 2006 (*22*). Limited reports of IAV isolation from cattle exist; most reports occurred during the 1960s and 1970s in Hungary and in the former Soviet Union, where H3N2 was recovered from cattle experiencing respiratory disease (*9*,*23*). Direct detection of IAV in milk and the potential transmission from cattle to cats through feeding of unpasteurized milk has not been previously reported.

An IAV-associated drop in milk production in dairy cattle appears to have occurred during >4 distinct periods and within 3 widely separated geographic areas: 1949 in Japan (*17*), 1997–1998 and 2005–2006 in Europe (*19*,*21*), and 2024 in the United States (this report). The sporadic occurrence of clinical disease in dairy cattle worldwide might be the result of changes in subclinical infection rates and the presence or absence of sufficient baseline IAV antibodies in cattle to prevent infection. Milk IgG, lactoferrin, and conglutinin have also been suggested as host factors that might reduce susceptibility of bovids to IAV infection (*9*). Contemporary estimates of the seroprevalence of IAV antibodies in US cattle are not well described in the published literature. One retrospective serologic survey in the United States in the late 1990s showed 27% of serum samples had positive antibody titers and 31% had low-positive titers for IAV H1 subtype-specific antigen in cattle with no evidence of clinical infections (*24*). Antibody titers for H5 subtype-specific antigen have not been reported in US cattle.

The susceptibility of domestic cats to HPAI H5N1 is well-documented globally (*10*–*12*,*25*–*28*), and infection often results in neurologic signs in affected felids and other terrestrial mammals (*4*). Most cases in cats result from consuming infected wild birds or contaminated poultry products (*12*,*27*). The incubation period in cats is short; clinical disease is often observed 2–3 days after infection (*28*). Brain tissue has been suggested as the best diagnostic sample to confirm HPAI virus infection in cats (*10*), and our results support that finding. One unique finding in the cats from this report is the presence of blindness and microscopic lesions of chorioretinitis. Those results suggest that further investigation into potential ocular manifestations of HPAI H5N1 virus infection in cats might be warranted.

The genomic sequencing and subsequent analysis of clinical samples from both bovine and feline sources provided considerable insights. The HA and NA sequences derived from both bovine milk and cat tissue samples from different Texas farms had a notable degree of similarity. Those findings strongly suggest a shared origin for the viruses detected in the dairy cattle and cat tissues. Further research, case series investigations, and surveillance data are needed to better understand and inform measures to curtail the clinical effects, shedding, and spread of HPAI viruses among mammals. Although pasteurization of commercial milk mitigates risks for transmission to humans, a 2019 US consumer study showed that 4.4% of adults consumed raw milk >1 time during the previous year (*29*), indicating a need for public awareness of the potential presence of HPAI H5N1 viruses in raw milk.

Ingestion of feed contaminated with feces from wild birds infected with HPAI virus is presumed to be the most likely initial source of infection in the dairy farms. Although the exact source of the virus is unknown, migratory birds (Anseriformes and Charadriiformes) are likely sources because the Texas panhandle region lies in the Central Flyway, and those birds are the main natural reservoir for avian influenza viruses (*30*). HPAI H5N1 viruses are well adapted to domestic ducks and geese, and ducks appear to be a major reservoir (*31*); however, terns have also emerged as an important source of virus spread (*32*). The mode of transmission among infected cattle is also unknown; however, horizontal transmission has been suggested because disease developed in resident cattle herds in Michigan, Idaho, and Ohio farms that received infected cattle from the affected regions, and those cattle tested positive for HPAI H5N1 (*33*). Experimental studies are needed to decipher the transmission routes and pathogenesis (e.g., replication sites and movement) of the virus within infected cattle.

In conclusion, we showed that dairy cattle are susceptible to infection with HPAI H5N1 virus and can shed virus in milk and, therefore, might potentially transmit infection to other mammals via unpasteurized milk. A reduction in milk production and vague systemic illness were the most commonly reported clinical signs in affected cows, but neurologic signs and death rapidly developed in affected domestic cats. HPAI virus infection should be considered in dairy cattle when an unexpected and unexplained abrupt drop in feed intake and milk production occurs and for cats when rapid onset of neurologic signs and blindness develop. The recurring nature of global HPAI H5N1 virus outbreaks and detection of spillover events in a broad host range is concerning and suggests increasing virus adaptation in mammals. Surveillance of HPAI viruses in domestic production animals, including cattle, is needed to elucidate influenza virus evolution and ecology and prevent cross-species transmission.
